# Elucidating the Mechanisms Underlying Enhanced Drought Tolerance in Plants Mediated by Arbuscular Mycorrhizal Fungi

**DOI:** 10.3389/fmicb.2021.809473

**Published:** 2021-12-23

**Authors:** Shen Cheng, Ying-Ning Zou, Kamil Kuča, Abeer Hashem, Elsayed Fathi Abd_Allah, Qiang-Sheng Wu

**Affiliations:** ^1^College of Horticulture and Gardening, Yangtze University, Jingzhou, China; ^2^Department of Chemistry, Faculty of Science, University of Hradec Kralove, Hradec Kralove, Czechia; ^3^Department of Botany and Microbiology, College of Science, King Saud University, Riyadh, Saudi Arabia; ^4^Department of Plant Production, College of Food and Agricultural Sciences, King Saud University, Riyadh, Saudi Arabia

**Keywords:** drought tolerance, mycorrhizae, plant physiology, symbiosis, water deficit

## Abstract

Plants are often subjected to various environmental stresses during their life cycle, among which drought stress is perhaps the most significant abiotic stress limiting plant growth and development. Arbuscular mycorrhizal (AM) fungi, a group of beneficial soil fungi, can enhance the adaptability and tolerance of their host plants to drought stress after infecting plant roots and establishing a symbiotic association with their host plant. Therefore, AM fungi represent an eco-friendly strategy in sustainable agricultural systems. There is still a need, however, to better understand the complex mechanisms underlying AM fungi-mediated enhancement of plant drought tolerance to ensure their effective use. AM fungi establish well-developed, extraradical hyphae on root surfaces, and function in water absorption and the uptake and transfer of nutrients into host cells. Thus, they participate in the physiology of host plants through the function of specific genes encoded in their genome. AM fungi also modulate morphological adaptations and various physiological processes in host plants, that help to mitigate drought-induced injury and enhance drought tolerance. Several AM-specific host genes have been identified and reported to be responsible for conferring enhanced drought tolerance. This review provides an overview of the effect of drought stress on the diversity and activity of AM fungi, the symbiotic relationship that exists between AM fungi and host plants under drought stress conditions, elucidates the morphological, physiological, and molecular mechanisms underlying AM fungi-mediated enhanced drought tolerance in plants, and provides an outlook for future research.

## Introduction

Drought stress (DS) seriously impacts crop growth and productivity (He et al., 2020). Reduced rainfall and global warming are leading to frequent episodes of drought globally. DS decreases photosynthetic efficiency in plants, reduces assimilate production, and impairs cell structure and function (Liang et al., 2019). DS also induces the accumulation of reactive oxygen species (ROS) in plant cells, causing an oxidative burst, which results in protein denaturation and the degradation of cell membranes (Bahadur et al., 2019). The balance of endogenous hormones in plants is also disturbed by DS, triggering a negative response in plant growth and metabolism (Zhang H. Y. et al., 2018).

The plant rhizosphere is inhabited by numerous, diverse microorganisms including bacteria and fungi. Notably, arbuscular mycorrhizal (AM) fungi have evolved to become symbionts with most terrestrial plants (He et al., 2019). AM fungi infect and reside within and on the surface of plant roots and are involved in the absorption of water and nutrients used by the host plant in exchange for carbohydrates that are provided by their host plant (Jiang et al., 2017). AM fungi dramatically improve the tolerance of host plants to abiotic and biotic stresses (Li et al., 2016). The promotional effect of AM fungi on plant growth is attributed to their developed mycelium network and to glomalin production (Latef et al., 2016). The enhancement of DS by AM fungi is associated with osmotic adjustment, antioxidant defense system, polyamines (PAs), fatty acids (FAs), mineral nutrition acquisition, and induction of gene expression in host plants (Wu et al., 2013; Cheng et al., 2020; Zhang et al., 2020). AM symbiosis alters the physiology of host plants under DS conditions, including adjustments in water potential and gas exchange (Gholamhoseini et al., 2013). This is due to the participation of extraradical hyphae in the absorption of water by mycorrhizal roots, and concomitant increases in the rate of photosynthesis (Zhang F. et al., 2018). The changes in water status of mycorrhizal plants under DS conditions is modulated by hormone signals that result in osmotic adjustments (Wu and Xia, 2006a).

The mechanisms that effectively contribute to enhanced drought tolerance in host plants by AM fungi are complex and involve multiple plant responses (Table 1), along with the mechanism of mycorrhizal fungi themselves. Therefore, the present review summarizes the mechanisms by which AMs enhance plant drought tolerance at the morphological, physiological, and molecular levels. Such review would highlight a theoretical basis to understand AM functions on stress tolerance and subsequently potential application of AM fungi in crops of arid areas.

**TABLE 1:** Effects of AM fungi inoculation on plant growth and physiological and molecular responses of host plants under drought stress in the selective literatures.

Drought responses	Plant species	AM fungal species	AM fungal variables	Plant variables	References
Plant growth	*Ephedra foliata*	Mixture of *Claroideoglomus etunicatum*, *Funneliformis mosseae*, and *Rhizophagus intraradices*	Total col%^↓^	Shoot fresh and dry weight^↑^; Root length^↑^; Root fresh and dry weight^↑^	Al-Arjani et al., 2020
	*Glycine max*	No specific AM fungi mentioned	Col%^↑^	LAI^↑^; growth performance^↑^	Pavithra and Yapa, 2018
	*Ipomoea batatas*	*Glomus* sp. and *Acaulospora* sp.	Col%*^ns^*	Plant growth^↑^; tubers per plant^↑^; tuber weight^↑^	Yooyongwech et al., 2016
	*Panicum turgidum*	Mixture of *C*. *etunicatum*, *F*. *mosseae*, and *R*. *intraradices*	Total col%^↓^	Root morphology (length, surface area and volume)^↑^	Abd_Allah et al., 2019
	*Phoenix dactylifera*	*G*. *clarum*, *G*. *deserticola*, and *G*. *monosporus*	Col%^↑^	Leaf number^↑^; LAI^↑^	Meddich et al., 2015
	*Poncirus trifoliata*	*Diversispora versiformis*	Col%^↓^; soil hyphae length^↑^	Plant growth performance^↑^; number of lateral roots^↑^; root morphology^↑^	Zou et al., 2017
	*Poncirus trifoliata*	*C. etunicatum*, *D*. *versiformis*, *F. mosseae*, and *Rhizoglomus intraradices*	Col%^↓^	Plant height^↑^; shoot and root biomass^↑^; root hairs density^↑^; leaf number^↑^; stem diameter^↑^	Liu C. Y. et al., 2018
Soil and plant nutrient	*Citrus tangerina*	*G*. *etunicatum* and *G*. *mosseae*	Col%^↓^; Hyphal length^↓^	Soil moisture^↑^; soil water potential^↑^	Zou et al., 2013
	*Solanum lycopersicum cv.*	*F*. *mosseae*, and *R*. *intraradices*	Col%*^ns^*	leaf P content^↑^; *LePT3*^↑^; *LePT4*^↑^; *LePT5*^↑^;	Volpe et al., 2018
	*Ephedra foliata*	Mixture of *C*. *etunicatum*, *F*. *mosseae*, and *R*. *intraradices*	Total col%^↓^	Ammonium^↑^; nitrate^↑^; nitrate reductase^↑^; nitrite reductase^↑^	Al-Arjani et al., 2020
	*Medicago sativa*	*Acaulospora scrobiculata*, *G*. *intraradices*, and *D*. *spurcum*	Col%^↓^	Soil structure^↑^; P^↑^	Ye et al., 2013
	*Ipomoea aquatica*	Unidentified AM fungi	Col%^↑^	Mineral nutrients (P, K, Mg, Na, Fe, Mn, and Zn) uptake^↑^	Halder et al., 2015
	*Salsola laricina*	*Archaeospora schenckii*, *Scutellospora erythropa*, *Septoglomus deserticola*, and *Septoglomus constrictum*	Col%^↓^	P uptake^↑^	Nouri et al., 2020
	*Vaccinium* sp.	*G*. *clarum*, *G*. *etunicatum*, *Gigaspora margarita*, and *Scutellospora heterogama*	Col%^↑^	Nutrient absorption^↑^	Farias et al., 2014
	*Poncirus trifoliata*	*G*. *versiforme*	Col%^↓^	N^↑^; K^↑^; Ca^↑^; Fe^↑^	Wu and Zou, 2009
Photosynthesis	*Ephedra foliata*	Mixture of *C*. *etunicatum*, *F*. *mosseae*, and *R*. *intraradices*	Total col%^↓^	Chlorophyll a^↑^, b^↑^, and a + b^↑^	Al-Arjani et al., 2020
	*Ipomoea batatas*	*Glomus* sp. and *Acaulospora* sp.	Col%*^ns^*	Chlorophyll degradation^↓^; photosynthetic pigments^↑^; maximum quantum yield of PSII (Fv/Fm); photon yield of PSII (ΦPSII) ^↑^; Pn^↑^	Yooyongwech et al., 2016
	*Leymus chinensis*	G. mosseae	Col%^↑^	Gs^↑^; Pn^↑^; chlorophyll^↑^	Lin et al., 2017
	*Panicum turgidum*	*Mixture of* C. etunicatum, F. mosseae, *and* R. intraradices	Total col%^↓^	Chlorophyll a^↑^, b^↑^, and a + b^↑^; E^↑^; Gs^↑^;	Abd_Allah et al., 2019
	*Ricinus communis*	F. mosseae *and* Rhizophagus intraradices	Col%*^ns^*	Gs^↑^; Pn^↑^; E^↑^; Ci^↓^; Chlorophyll a^↑^, b^↑^, and a + b^↑^	Zhang T. et al., 2018
	*Robinia pseudoacacia*	*R*. *irregularis* and *G*. *versiforme*	Col%^↑^	Chlorophyll^↑^; Pn^↑^; effective quantum yield of PSII(ΦPSII) ^↑^; calorific value^↑^; carbon^↑^	Zhu et al., 2014
	*Zea mays*	*R. irregularis*	Col%*^ns^*	Pn^↑^; Gs^↑^; Ci*^ns^*	Quiroga et al., 2019
	*Sophora davidii*	*G*. *constrictum* and *G*. *mosseae*	Col%^↑^	Gs^↑^; Pn^↑^; Ci^↓^; maximum quantum yield of PSII (Fv/Fm) ^↑^	Gong et al., 2013
Antioxidant responses	*Celtis caucasica*	*F*. *mosseae* and *R*. *intraradices*	Col%^↑^	CAT^↑^; SOD^↑^; MDA^↓^; H_2_O_2_^↓^	Sepahvand et al., 2021
	*Ephedra foliata*	Mixture of *C*. *etunicatum*, *F*. *mosseae*, and *R*. *intraradices*	Total col%^↓^	MDA^↓^; H_2_O_2_^↓^; SOD^↑^; CAT^↑^; APX^↑^; GPX^↑^; glutathione reductase^↑^; reduced glutathione^↑^; ascorbic acid^↑^	Al-Arjani et al., 2020
	*Leymus chinensis* and *Hemarthria altissima*	*Glomus* sp.	Col%^↑^	CAT^↑^; SOD^↑^; MDA^↓^	Li et al., 2019
Polyamine metabolism	*Poncirus trifoliata*	*F. mosseae*	Col%^↑^	Put and Cad^↑^; Spd and Spm^↑^; PA catabolic enzyme activity^↑^; Put-synthases^↑^	Zhang et al., 2020
	*Pelargonium graveolens*	*G*. *intraradices* and *G*. *mosseae*	Col%^↑^	Essential oil^↑^; total phenol^↑^; flavonoids^↑^; CAT^↑^; SOD^↑^; APX^↑^; GPX^↑^; MDA^↓^; H_2_O_2_^↑^	Amiri et al., 2015
	*Poncirus trifoliata*	*F. mosseae*	Col%^↑^	O_2_^–↓↓^; H_2_O_2_^↓^; MDA^↑^	Zou et al., 2021b
Osmotic adjustment	*Poncirus trifoliata*	*F*. *mosseae* and *Paraglomus occultum*	Col%^↓^	Sucrose, glucose and fructose^↑^; proline^↓^	Wu et al., 2017
	*Erythrina variegat*	*G. mosseae*	Col%^↓^	Total soluble sugar^↓^; protein^↑^; proline^↓^	Manoharan et al., 2010
	*Ephedra foliata*	Mixture of *C. etunicatum, F. mosseae*, and *R. intraradices*	Total col%^↓^	Glucose^↑^; proline^↑^; soluble protein^↑^	Al-Arjani et al., 2020
	*Populus canadensis*	*R. irregularis*	Col%^↑^	Free proline^↓^; soluble protein^↑^	Liu T. et al., 2016
	*Ricinus communis*	*F. mosseae* and *R. intraradices*	Col%*^ns^*	Free proline^↑^; soluble protein^↑^	Zhang T. et al., 2018
	*Zenia insignis*	*D. versiformis, F. mosseae*, and *R. intraradices*	Col%*^ns^*	Proline^↑^; soluble sugars^↑^	Zhang Z. et al., 2018
	*Triticum aestivum*	*Rhizoglomus irregulare*	Col%^↓^	Proline*^ns^*	Pons et al., 2020
	*Macadamia tetraphylla*	*Acaulospora* sp., *Glomus* sp., *Gigaspora* sp., and *Scutellospora* sp.	Col%^↑^	Proline^↑^; soluble sugars^↑^	Yooyongwech et al., 2013
	*Poncirus trifoliata*	*F. mosseae*	Col%^↑^	Put^↑^; Cad^↑^; Spd^↑^; ADC^↑^; ODC^↑^; SPMS^↓^; SPDS^↑^; DAO^↑^; PAO^↑^; Precursor of PA^↑^	Zou et al., 2021b
	*Poncirus trifoliata*	*G. mosseae*	Col%^↑^	Put and Spd^↓^; Spm^↑^; SPMS^↑^	Luo, 2009
	*Zea mays*	*R. irregularis*	Col%*^ns^*	Put*^ns^*; ODC and GABA^↓^; DAO^↑^	Hu et al., 2020
	*Zea mays*	*R. irregularis*	Col%^↑^	Put^↓^; DAO and GABAT^↑^; GABA^↑^	Hu and Chen, 2020
Fatty acids	*Glycine max*	*Claroideoglomus etunicatum*, *F. mosseae, Gigaspora gigantea, R. clarus*, and *Paraglomus occultum*	Col%^↑^	MUFA^↑^; PUFA^↑^	Igiehon et al., 2021
	*Poncirus trifoliata*	*F. mosseae*	Col%^↓^	UFA^↑^; SFA^↓^; *PtFAD2*^↑^; *PtFAD6*^↑^; *Pt*Δ*9*^↑^;*Pt*Δ*15*	Wu et al., 2019
	*Sesamum indicum*	*F. mosseae* and *R. irregularis*	Col%^↑^	UFA^↑^; SFA^↓^	Gholinezhad and Darvishzadeh, 2021
Endogenous hormones	*Poncirus trifoliata*	*D. versiformis*	Col%^↓^	ABA^↑^; GA^↑^; IAA^↑^; MeJA^↑^; ZR^↑^	Zhang et al., 2017
	*Solanum lycopersicum*	*F. mosseae* and *R. intraradices*	Col%^↑^	ABA^↑^	Chitarra et al., 2016
	*S*. *lycopersicum*	*R. irregularis*	Col%*^ns^*	ABA^↓^; IAA^↑^; MeJ^↑^; SA^↑^	Sanchez-Romera et al., 2018
	*Ephedra foliata*	Mixture of *C. etunicatum*, *F. mosseae*, and *R. intraradices*	Total col%^↓^	ABA^↑^; IAA^↑^; IBA^↑^; GA^↑^	Al-Arjani et al., 2020
	*Glycyrrhiza uralensis*	*R. irregularis*	Col%^↓^	ABA^↓^	Xie et al., 2018
	*Panicum turgidum*	Mixture of *C. etunicatum, F. mosseae*, and *R. intraradices*	Total col%^↓^	IAA^↑^	Abd_Allah et al., 2019
	*Zea mays*	*G. manihotis*		ABA^↓^	Kandowangko et al., 2009
Molecular regulation	*Glycine max* and *Lactuca sativa*	*G. mosseae* and *G. intraradices*	Col%*^ns^*	*GmPIP2*^↓^; *LsPIP1*^↓^; *LsPIP2*^↓^	Porcel et al., 2006
	*Zea mays*	*G. intraradices*	Col%^↑^	*GintAQPF1*^↑^; *GintAQPF2*^↑^; AQP activities^↑^	Li et al., 2013
	*Poncirus trifoliata*	*F. mosseae*	Col%^↓^	*PtAHA2*^↑^; H^+^-ATPase activity^↑^	Cheng H. Q. et al., 2021
	*Malus domestica*	*R. irregularis*	Col%^↑^	Strigolactone^↑^; strigolactone synthesis genes expression^↑^; *MdIAA24*^↑^	Huang et al., 2021a
	*Zea mays*	*R. intraradices*	Col%^↑^	*ZmPIP1;1*^↓^; *ZmPIP1;3*^↓^; *ZmPIP1;4*^↓^; *ZmPIP1;6*^↓^; *ZmPIP2;2*^↓^; Z*mPIP2;4*^↓^; *ZmTIP1;2*^↓^; ZmPIP2; 5 protein content^↑^	Bárzana et al., 2014
	*Helianthemum almeriense*	*Terfezia claveryi*	Col%^↑^	*HaPIP1;1* ^↓^	Navarro-Ródenas et al., 2013

^↑^ and ^↓^ indicate the significant increasing and decreasing response of the variable to mycorrhizal fungal inoculation. ns, no significant difference; AM, arbuscular mycorrhiza; Col, AM fungal colonization; LAI, leaf area index; Gs, stomatal conductance; Pn, net photosynthetic rate; Ci, intercellular CO_2_ concentration; E, transpiration rate; CAT, catalase; SOD superoxide dismutase; MDA, malondialdehyde; H_2_O_2_, hydrogen peroxide; APX, ascorbate peroxidase; GPX, glutathione peroxidase; O_2_^–^, superoxide radical; Put, putrescine; Cad, cadaverine; Spd, spermidine; Spm, spermine; ADC, arginine decarboxylase; ODC, ornithine decarboxylase; SPMS, spermine synthase; SPDS, spermidine synthase; DAO, diamine oxidase; PAO, polyamine oxidases; SPDS, spermidine synthetase; GABAT, γ-aminobutyrate aminotransferase; GABA, γ-aminobutyric acid; MUFA, monounsaturated fatty acid; PUFA, polyunsaturated fatty acid; UFA, unsaturated fatty acid; SFA, saturated fatty acid; ABA, abscisic acid; GA, gibberellin; IAA, indoleacetic acid; MeJA, methyl jasmonate; ZR, zeatin nucleoside; SA, salicylic acid.

## Impact of Drought Stress on Arbuscular Mycorrhizal Fungal Diversity

Arbuscular mycorrhizal fungi are abundant, widely distributed, and adaptable to a variety of ecological environments where they contribute to several ecological processes, including enhancing the stress tolerance of host plants (Chen et al., 2018). The diversity of AM fungi present in a soil environment can vary, depending on the species of host plants present, soil types, and environmental conditions (Lenoir et al., 2016). Over 244 species of Glomeromycota have been identified in a variety of ecosystems (van der Heijden et al., 2015). Low species diversity in AM symbiosis when there is high selectivity by the plant host on their fungal symbiont (Egger and Hibbett, 2004). Functional differences among strains of AM fungi also contribute to low AM fungal diversity by further influencing the network of functional AM fungi and host plants from species to specific AM genotypes (Schüβler et al., 2001). AM fungi play an essential role in improving the productivity of ecosystems and maintaining an ecological balance, especially where AM fungi help host plants to survive in an arid environment (Mathur et al., 2019). Soil water deficits, however, also reduce the colonization ability of AM fungi, hyphal elongation, and spore germination (Wu and Zou, 2017; Zhang F. et al., 2018).

Arbuscular mycorrhizal fungal diversity in water-deficient soils is comparatively lower than it is in water-saturated soils, as water deficit negatively impacts the diversity of AM fungi. However, in watermelon, the 18S rRNA copy numbers of AM fungi were increased in AM versus non-AM roots under DS, indicating that under DS conditions, exogenous AM fungi increase native fungal diversity under water deficit conditions only, resulting in improved colonization and plant responses (Omirou et al., 2013). Although AM fungi are sensitive to droughted soils, individual strains or isolates of AM fungi commonly exist in these environments that are tolerant to DS (Wu et al., 2013). Native strains of AM fungi present in arid environments exhibit a long-term adaptation to dry soils (Sylvia and Williams, 1992). For example, *Glomus* species are commonly present in semi-arid ecosystems, and survive and grow under conditions of low-water availability (Omirou et al., 2013). The AM fungal species *Funneliformis mosseae* can tolerate different environmental conditions, including DS, and is thus considered an early stage colonizer of plants (Lenoir et al., 2016). An antagonistic interaction can exist, however, between host plants and AM fungi diversity under drought conditions. For example, low richness of barley species results in high AM fungal richness under drought (Sendek et al., 2019).

## Effect of Drought Stress on Mycorrhizal Symbiosis

Drought stress strongly inhibits crop growth, although plants have evolved several strategies to enhance DS tolerance and resistance. Plant roots have a high plasticity and have evolved a symbiotic relationship with AM fungi that enhances plant nutrient uptake and water acquisition under DS conditions (Zou et al., 2017). As a result, AM fungi promote crop growth under adverse conditions such as DS (Begum et al., 2019). The growth and development of AM fungi require a certain level of soil moisture, and the level of soil moisture condition strongly affects spore germination, hyphal growth, hyphal branching, and the formation of secondary spores (Omirou et al., 2013; Wu et al., 2013). Therefore, AM fungi must invest more resources in the storage capacity of roots to tolerate a DS environment (Lenoir et al., 2016). AM fungi are an important component of the drought resistance of plants growing in desert ecosystems (Vasar et al., 2021). Water-deficient soils, however, can limit the development of AM fungi in the soil and the rhizosphere, although some AM fungal species can adapt to dry soils and still maintain a relatively high level of root colonization, which is essential for the survival and growth of host plants (Zou et al., 2017). After analyzing the results of a large number of studies, Augé (2001) reported on the diverse effects of drought on AM fungal colonization, manifested as a decrease, undetectable change, or an increase in colonization under DS conditions. Surprisingly, when certain plants experience DS, they secret rhizospheric signaling molecules to attract AM fungi (Oldroyd, 2013). AM fungi also have a certain level of intrinsic drought tolerance, which is imparted to AM host plants under drought conditions. Mycorrhizal spores are resistant to and can survive under drought conditions, thus ensuring a source of AM fungi for continued infection of plant roots (Song, 2005). Notably, soil drought was not a greater determinant of mycorrhizal species or colonization rate than low-temperature, as root growth is more affected by low-temperature than by water-deficient soils (Kilpelainen et al., 2020b). Therefore, poor performance of AM under drought conditions implies an avoidance of DS rather than a tolerance of DS (Kilpelainen et al., 2020b). Importantly, AM fungi isolated from unfavorable environments have been reported to be more effective in enhancing plant stress tolerance (Rivero et al., 2018). Under extreme soil drying, co-adaption of local plants and local soil AM fungi would induce the abundance of mycorrhizal hyphae and arbuscules and fewer vesicles than under moderate soil drying, thus, mitigating DS (Remke et al., 2021). Future studies, however, will need to further analyze how soil moisture levels affect root colonization by mycorrhizal fungi and the acquisition of soil nutrients, such as N and P content, as well as pH.

## Drought-Stress Adaptive Mechanisms Induced in Plants by Arbuscular Mycorrhizal Fungi

**FIGURE 1:**
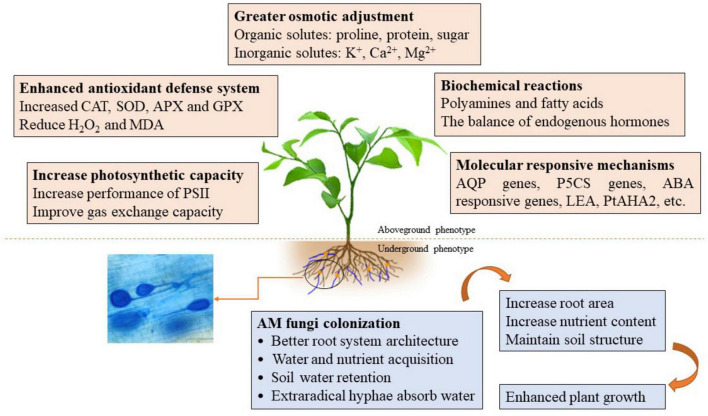
A schematic diagram regarding the underlying mechanisms of AM fungi enhancing plant drought tolerance. AM fungi affect the morphology, physiological activities, and molecular regulation of host plants through direct or indirect interactions in response to drought stress, and thus help host plants regulating and maintaining various processes to deal with the harmful effects of drought on plant.

Drought stress has become the major abiotic stress limiting crop growth and productivity (Bárzana and Carvaja, 2020). AM fungi, however, can mitigate the unfavorable effects of DS on plant growth by a series of mechanisms (Pavithra and Yapa, 2018). AM-enhanced drought tolerance of host plants, however, is a complex process, shaped by both the AM fungal species and the plant host. The mechanisms involved in morphological adaptability and physiological and molecular responses had been described by Wu and Zou (2017) and Bahadur et al. (2019). Here, we focused on the recent advances in mycorrhizal regulation of host polyamines and FAs, as well as the expression of stressed genes in arid environments (Table 1 and Figure 1).

### Morphological Adaptations in Host Plants and Arbuscular Mycorrhizal Fungi

Arbuscular mycorrhizal symbiosis is associated with morphological adaptations to DS in host plants that improve tolerance to arid environments (Ruiz-Lozano and Aroca, 2010a). AM fungi have been reported to induce DS-tolerant growth phenotypes in response to DS in a variety of plant plants, including trifoliate orange, date palm, and soybean (Grümberg et al., 2015; Meddich et al., 2015; Zou et al., 2017; Table 1). Although DS has a strong inhibitory effect on root system growth and development, inoculation of root systems with AM fungi significantly reduce this negative impact (Wu and Xia, 2006b). AM fungi affect the penetration and distribution of roots in soil by changing root morphology in host plants (Liu J. et al., 2016). Studies have shown significant increases in root traits, such as biomass, length, volume, and surface area, as well as root-hair density, root-hair length, and root branching, occur in trifoliate orange under DS conditions after AM fungal colonization (Zou et al., 2017; Liu C. Y. et al., 2018; Zhang F. et al., 2018; Table 1). This implies that increases in water and nutrients occur by optimizing root morphology in response to DS. In addition, mycorrhizal plants maintain their water balance by altering their degree of defoliation (Bryla and Duniway, 2010).

In addition to plant morphology, morphological changes in AM fungi also occur in response to DS conditions. DS negatively affects spore germination and subsequent hyphal development, thus interfering with the formation and further development of mycorrhizal structures, such as arbuscules and vesicles (Giovannetti et al., 2010). Interestingly spores of *Glomus mosseae* and *G*. *deserticola* had greater colonization ability after storage at a –0.04 MPa soil water potential, while *G*. *fasciculatum* was most infectious after storage at a –0.8 MPa soil water potential (Wu et al., 2013), indicating that the moisture level in the soil surrounding fungal spores affects the colonization of AM fungi. It also suggests that AM fungi exhibit morphological and ecological adaptability to DS.

### Arbuscular Mycorrhizal-Mediated Water and Nutrient Acquisition

A functional aspect of AMs is their ability to increase water and nutrient acquisition in host plants under DS conditions through the production of mycorrhizal extraradical hyphae (Zhao et al., 2015). Mycorrhizae extend from their host plants and form a developed hyphal network in the surrounding soil that partially reduces the flow resistance between the host plant and the surrounding soil (Allen, 2007), thus, improving the water use efficiency of host plants under DS conditions. The size of air spaces between plant roots and the soil particles increase under DS conditions and mycorrhizal hyphae with the diameter of 2–5 μm can bridge the gap between the roots and soil to help maintain the continuity of water transport. Mycorrhizal hyphae absorb water at a higher rate under DS conditions than they do under sufficient water conditions, reflecting their benefit to host plants growing in arid environmental conditions (Zhang F. et al., 2018). Nevertheless, plants obtain less water through AM fungal hyphae, relative to the overall water absorption by roots and the overall water demand of plants (Püschel et al., 2020). In addition, the presence of common mycorrhizal networks among different plants can redistribute interspecific nutrients under DS conditions, thus realizing the enhancement of drought tolerance of intercropped crops in an arid environment (Mickan et al., 2021).

Mycorrhizal symbionts promote nutrient acquisition in their host plants subjected to drought conditions (Table 1). For example, inoculation of trifoliate orange plants with *G*. *versiforme* increased the level of P, K, Ca, and Fe in leaves and roots under both well-watered and DS conditions (Wu and Zou, 2009). Leaf N, P, and K content also increased in mycorrhizal blueberry plants exposed to DS (Farias et al., 2014). Notably, AM fungi increase both the availability of soil P and the host plant’s ability to absorb P, along with increasing the uptake of K, Mg, Fe, Mn, and Zn under DS conditions (Halder et al., 2015). Püschel et al. (2021) further using two-compartment rhizoboxes reported that mycorrhizal P uptake was more effective over relatively short distances (i.e., near plant roots) and less effective over relatively long distances (i.e., exclusively by mycorrhizal hyphal transport) under DS conditions. The long distance mycorrhizal pathway, however, still offered significant advantages in long-distance P transport, especially under medium and low moisture conditions, with a concomitant indirect effect of AM fungi changing substrate hydraulic conductivity (Püschel et al., 2021). Inoculation of plant roots with AM fungi also significantly contributed to P uptake in *Salsola laricina*, a species which is commonly planted for the restoration of degraded grassland (Nouri et al., 2020). The higher P content in AM plants under DS conditions is a primary reason for their greater tolerance to drought stress relative to non-AM plants (Kilpelainen et al., 2020a). The PHOSPHATE TRANSPORTER1 (PHT1) gene family is involved in the uptake and translocation of phosphate (Pi) in the soil, as well as the uptake of Pi from AM (Liu F. et al., 2018). AM fungi induced phosphate transporter (PT) genes expression (*LePT4* and *LePT5*) in tomato plants to enhance tolerance to soil water deficit, dependent on the fungal species (Volpe et al., 2018). Likewise, the expression of certain host *PT* genes (e.g., *PHT1.2* and *PHO9* in *Populus trichocarpa*) was increased under drought conditions, independent of Pi levels, while other *PT* genes may be Pi-dependent (Zhang C. X. et al., 2016). In addition, ammonium transporter protein and potassium (K^+^) transporter genes collectively were up-regulated by AM fungal inoculation under DS (Balestrini et al., 2019), which is critical for N and K uptake of host plants in arid environment. Interestingly, drought-adapted AM fungal strains represent better improved nutrient contents of the host plant (i.e., lavender) in arid environments than non-adapted AM fungal strains (Marulanda et al., 2007; Symanczik et al., 2018). In addition, the activities of H^+^-ATPase and Ca^2+^-ATPase of AM extraradical hyphae were induced under DS and caused the acidification of soil environment, which facilitates the absorption of mineral nutrients and the signal exchange between AM fungi and plants to enhance the drought tolerance of plants (Ferrol et al., 2000; Xu et al., 2018a).

Higher water and nutrient uptake of mycorrhizal plants versus non-mycorrhizal plants is also associated with changes in root architecture, including the production of root hairs (Liu C. Y. et al., 2018). Cheng H. Q. et al. (2021) recently reported on the expression of a *PtAHA2* gene, which is involved in H^+^-ATPase activity, in trifoliate orange, which was expressed in leaf and root tissues under both well-watered and DS conditions, after plants were inoculated with *F*. *mosseae.* Such expression patterns in mycorrhizal plants under DS conditions could cause an acidic rhizospheric microenvironment, thus, leading to an increase in NH_4_^+^ in leaves and roots of AM plants. AM symbiosis also induces the expression of aquaporin (*ZmTIP1;1*) gene expression at high NH_4_^+^ concentrations, resulting in excessive N storage in vacuoles under DS conditions (Quiroga et al., 2020a). Transcriptomic studies also revealed that under DS, nutrient transporters in AM plants, such as *PT*, ammonium transporter, potassium (K^+^) transporter, amino acid transporter, peptide transporter and sulfate transporter, were significantly up-regulated by mycorrhizal fungi (Balestrini et al., 2019). This indicates that the improvement of nutrient absorption by mycorrhizas is closely linked to the regulation of nutrient transporters under DS conditions.

### Arbuscular Mycorrhizal-Improved Soil Aggregate Formation

The formation of soil aggregates has a major impact on the saturation status of soils that directly impact plant growth and development (Vergani and Graf, 2016). Notably, AM fungi produce and secrete glomalin through mycelia and spores which adheres to the soil like “super glue” and contributes to the maintenance of good soil structure (Chi et al., 2018). Zou et al. (2014) reported that glomalin-related soil protein (GRSP), more specifically total GRSP but not easily extractable GRSP, contributed to soil water content, suggesting the importance of total GRSP in mediating soil aggregate stability and soil moisture. Mycorrhizal fungi alter the soil water cycle through their network of extraradical mycelia that promotes the formation of soil aggregates and increases soil water holding capacity (Querejeta et al., 2003). Augé et al. (2001) reported that hyphae and exudates of AM fungi improved soil structure by altering soil aggregates, thereby enhancing soil water retention, except for differences caused by root growth in mycorrhizal and non-mycorrhizal soils. In addition, AM soils can influence the growth of non-mycorrhizal plants, and the number of mycorrhizal hyphae in the soil differs significantly between non-AM and AM soils (Augé et al., 2004). Mycorrhizal extraradical hyphae also directly contribute to the dispersive energy in the formation of soil macroaggregates under DS conditions (Ji et al., 2019). Inoculation of *Medicago sativa* plants with AM fungi accelerated the formation of large soil aggregates and the stability of water-stable aggregates under DS conditions, thus, improving soil structure (Ye et al., 2013). Collective studies indicate that the water content of plants increases in mycorrhizal substrates under both water-saturated and DS soil conditions (Bitterlich et al., 2018). AM hyphae in soil may have the function of reducing the air gap at the soil-root interface, and thus better soil-root contact in AM soils promotes the soil-root hydraulic conductance (Augé, 2004). Hydraulic conductivity has been shown to be higher in mycorrhizal soil prior to and during DS conditions (Bitterlich et al., 2018).

### Arbuscular Mycorrhizal-Improved Photosynthetic Capacity

Solar radiation is essential for plant growth and development. DS induces stomatal closure, chloroplast structural damage, including the structure and function of the PSII reaction center, and the inhibition of electron transport (Zhang Y. M. et al., 2016). AM fungi have been reported to improve photosynthetic capacity in host plants under DS conditions, as indicated by the increase in net photosynthetic rates, transpiration rates, and stomatal conductance, as well as by a decrease in intercellular CO_2_ concentration (Table 1; Zhu et al., 2014; Lin et al., 2017; Zhang T. et al., 2018; Abd_Allah et al., 2019). Colonization of plants by AM fungi help the host plant to maintain the integrity and stability of both PSI and PSII under DS conditions (Mathur et al., 2019). This benefit is derived from the AM-enhancement of water absorption and transport and the stimulation of C sinks. AM fungi also directly or indirectly enhance photosynthetic efficiency and chlorophyll concentration under DS conditions (Gong et al., 2013; Zhang T. et al., 2018; Abd_Allah et al., 2019). AM fungi were reported to induce the expression of 14-3-3 genes that reduce the rate of transpiration and modulate stomatal behavior, both of which play a role in the maintenance of water use efficiency (Xu et al., 2018b). A study of AM plants subjected to rewatering of plants after exposure to DS condition demonstrated that AM plants were able to repair damaged photosynthesis-related structures and restore normal levels of photosynthesis faster than non-AM plants (Polcyn et al., 2019). The enhancement of photosynthetic capacity was also reported to be associated with a reduction of specific leaf area in AM plants (Quiroga et al., 2019) and an increase of Rubisco activity and electron transport rates (Valentine et al., 2006).

### Arbuscular Mycorrhizal-Enhanced Plant Host Antioxidant Defense Systems

Exposure of plants to DS induces an excessive accumulation of ROS, which results in oxidative damage to proteins, nucleic acids, and lipids (Hussain et al., 2019). Plant exposed to DS conditions activate both enzymatic and non-enzymatic antioxidant defense systems to remove excessive ROS in cells. Antioxidant enzymes include superoxide dismutase (SOD), peroxidase (POD), catalase (CAT), ascorbate peroxidase (APX), glutathione peroxidase (GPX), etc., while antioxidants include ascorbic acid (ASC), glutathione (GSH), flavonoids, carotenoids, etc. (Rapparini and Peñuelas, 2014). Antioxidant compounds directly eliminate ROS and also induce a series of signaling pathways that indirectly regulate ROS production in plant cells. Studies have demonstrated that AM symbiosis helps plants cope with the excessive accumulation of ROS in cells caused by drought stress by enhancing the host plant’s antioxidant defense systems, which reduces the oxidative damage that occurs in plant cells under DS conditions (Table 1; Rani et al., 2018; Zou et al., 2021a). Plants inoculated with AM fungi and exposed to DS conditions exhibit significantly enhanced levels of CAT, SOD, APX, and GPX activity, along with a reduction in H_2_O_2_ and malondialdehyde (MDA) content (Amiri et al., 2015; Al-Arjani et al., 2020). AM fungi-inoculated wheat plants were reported to have higher antioxidant enzyme activity and lower superoxide radical, H_2_O_2_, and MDA levels than non-inoculated plants exposed to DS in both drought-tolerant and drought-susceptible varieties (Rani et al., 2018). Mycorrhizal plants also exhibit higher levels of non-enzymatic antioxidants (GSH, ASC, and flavonoids) under DS conditions than non-mycorrhizal plants, which represents another mechanism to prevent DS-induced oxidative damage in host plants (Al-Arjani et al., 2020; Zou et al., 2021a). AM enhancement of antioxidant defense systems may be due to the accumulation of ROS in AM fungi themselves (Fester and Hause, 2005). In this regard, SOD genes have been identified in AM fungi (Corradi et al., 2009). Native AM fungi have been shown to exhibit higher antioxidant levels and AM development than non-native strains of AM fungi (Marulanda et al., 2007), suggesting that local, native strains of AM fungi should be explored for their potential use in agricultural production systems.

### Arbuscular Mycorrhizal-Mediated Osmotic Adjustments

Osmotic adjustments occur in response to drought conditions that involve both inorganic solutes (K^+^, Ca^2+^, Mg^2+^, etc.) and organic solutes (proline, sugar, proteins, glycine, etc.) (Ozturk et al., 2021). AM symbiosis improves the ability of host plants to make osmotic adjustments under DS conditions due to a greater accumulation of solutes in AM plants (Figure 1; Zou et al., 2021b). For example, AM fungi have been reported to improve the water status of sweet potato plants through the accumulation of AM fungi-regulated soluble sugars and free proline (Yooyongwech et al., 2016). Soluble sugars can function as a signal molecule that activates regulatory pathways controlling growth and development in plants and the transport of photosynthetic products (Poór et al., 2019). AM fungi substantially increase sucrose, fructose, and glucose concentrations in trifoliate orange and glucose concentrations in *Ephedra foliata* under DS conditions (Wu et al., 2017; Al-Arjani et al., 2020). These compounds can protect and stabilize macromolecule structures and maintain an appropriate water balance under DS conditions, thus alleviating DS-induced injury. In contrast, AM fungi reduced soluble sugar levels in *Erythrina variegata* under DS conditions (Manoharan et al., 2010), which perhaps could be due to the fact that AM plants exhibit less drought injury without the accumulation of solutes and sugars. In water-deficited plants, proline often functions as an osmoprotectant in DS plants that protects enzymes and proteins from denaturation (AlKahtani et al., 2021). Earlier studies reported lower proline content in mycorrhizal plants under DS conditions than in non-mycorrhizal plants (Manoharan et al., 2010; Liu J. et al., 2016; Wu et al., 2017). This again may be due to the fact that mycorrhizal plants are less affected by DS (Asrar et al., 2012; Pavla et al., 2013; Liu T. et al., 2016). It appears that AM-mediated reduction of proline in plants is caused by the inhibition of the proline synthesis pathway and the promotion of proline degradation (Zou et al., 2013). Other studies, however, have reported that AM plants exhibit a higher accumulation of proline under DS conditions than non-AM plants (Yooyongwech et al., 2013; Zhang T. et al., 2018; Zhang Z. et al., 2018; Al-Arjani et al., 2020). The accumulation of proline may provide energy and improve osmotic regulation in AM plants under DS conditions which would help to enhance growth (Yooyongwech et al., 2013). In contrast, it has also been shown that AM fungal inoculation of plants did not alter the proline content of wheat plants under DS conditions (Pons et al., 2020), indicating that mycorrhizal regulation of proline is influenced by the species of mycorrhizal fungus, the host plant, and the environment. In other words, AM-mediated changes in proline content are dependent upon host plants, AM fungi, and environmental conditions.

### Arbuscular Mycorrhizal-Mediated Polyamine Metabolism

Polyamines are low molecular weight polycationic compounds that are ubiquitous in living organisms (Ali et al., 2020b). Plant PAs mainly include cadaverine (Cad), putrescine (Put), spermine (Spm), and spermidine (Spd), which are associated with various stress responses in plants, but are also involved in many biological processes (Kateřina et al., 2019). PAs are involved in the plant-AM fungi interaction including mycorrhizal fungal colonization of root, mycorrhizal development, plant growth, and stress response (Table 1; Salloum et al., 2018). Studies revealed that exogenous application of PAs, especially Put, promotes the growth and development of AM fungi by stimulating AM fungal spore germination and primary hyphal elongation (Wu et al., 2010a; Yao et al., 2010). Endogenous PAs also regulate mycorrhizal development by altering the level of carbohydrates directed to roots (Wu et al., 2010b). In response to a persistent drought stress and 15-day water deficit conditions, trifoliate orange seedlings inoculated with *F*. *mosseae* had significantly higher levels of Put and Cad but reduced levels of Spd and Spm, relative to non-inoculated plants, in their roots (Zhang et al., 2020; Zou et al., 2021b). In maize, *Rhizophagus irregularis* inoculation also dramatically increased leaf Put concentrations in response to DS, along with more conversion of Put to γ-aminobutyric acid (GABA, a byproduct of PA degradation) (Hu et al., 2020). The activation of the synthesis of GABA by AM fungi may also contribute to enhanced DS tolerance in host plants by means of triggering stomatal closure (Hu and Chen, 2020; Hu et al., 2020). Mycorrhizal plants also had a higher ratio of (Spd + Spm)/Put under drought conditions, which prevented chlorophyll loss and a significant increase in the level of chlorophyll a (Zhang et al., 2020). The increase in PAs in response to DS also protects mycorrhizal plants against oxidative damage by maintaining cell pH and ion homeostasis and enhancing antioxidant defense systems (Zhang et al., 2020). The increment in (Spd + Spm)/Put in drought-stressed AM plants may be higher in sensitive genotype than in tolerant genotype (Sannazzaro et al., 2007). Mycorrhizae-induced increases in Put and Cad and decreases in Spm and Spd brought about by the presence of AM fungi could be attributed to an increase in level of PA precursors, such as arginine, ornithine, agmatine, and *S*-adenosyl methionine (Zou et al., 2021b), along with the down-regulation in the relative expression of PA catabolic enzyme genes (*CuAO* and *PAO*). The reduced levels of Spd and Spm in AM plants under DS conditions could also activate the signal associated with ROS, thus, up-regulating the expression of host antioxidant enzyme genes (e.g., *CAT* and *SOD*) (He et al., 2020; Zhang et al., 2020). Inoculation of plants with AM fungi, however, has also been reported to significantly reduce Put and Spd content in the leaves of host plants but increase Spm content (Luo, 2009; Hu and Chen, 2020). The decrease in Put and Spd levels in AM plants may be a result of the continuous conversion of Put and Spd to Spm in mycorrhizal plants to improve host drought tolerance (Luo, 2009). In addition, AM-induced changes in Put might accelerate trehalose synthesis of host plants, which is involved in improved osmotic adjustment as an organic compatible solute in response to stress (Garg and Saroy, 2020).

### Arbuscular Mycorrhizal-Mediated Fatty Acid Metabolism

The saturation level and composition of FAs in organisms are closely related to the lipid fluidity of the cell membrane, and a higher concentration of unsaturated fatty acids (UFAs) is associated with drought tolerance in plants (Mahnaz et al., 2020). FA metabolism has been implicated in AM regulation of plant drought response (Table 1). Trifoliate orange plants inoculated with *F*. *mosseae* had a higher UFA (e.g., C18:1, C18:2, and C18:3N3) level in roots, relative to non-inoculated plants, and a lower level of saturated fatty acids (SFA) (e.g., C18:0) under DS conditions, which resulted in a higher unsaturation index of FAs in AM plants compared to non-AM plants (Wu et al., 2019; Hu et al., 2020). *F*. *mosseae* and *Rhizophagus intraradices* also increased UFA contents and reduced SFA contents, in sesame plants, as well as increasing the level of non-enzymatic antioxidants under DS conditions (Gholinezhad and Darvishzadeh, 2021). Soybean plants inoculated with AM fungi combined with *Rhizobium cellulosilyticum* strain R3 also exhibited the highest percentage of UFAs, a feature that is beneficial to human health (Igiehon et al., 2021). AM plants also modulate changes in FA unsaturation by inducing the expression level of FA desaturase genes, such as FA desaturase 2 and FA desaturase 6 (Wu et al., 2019). The changes in the composition of FAs induced by AM fungi would help AM plants to maintain the fluidity of cell membranes, thus, mitigating the potential oxidative damage resulting from DS. On the other hand, AM fungi also dramatically increased C14:0 levels of host plants (Meng et al., 2021), which could favor the growth of budding spores and the formation of secondary spores, thus stimulating spore growth and subsequent hyphal colonization event (Sugiura et al., 2020). Such response in AM fungi will enable AM plants to better deal with the drought damage in dry environment than non-AM plants.

### Arbuscular Mycorrhizal-Regulation of Endogenous Hormones

Drought stress often induces changes in the level of endogenous hormone in plants (Liu T. et al., 2016). Studies have indicated that the levels of abscisic acid (ABA), indoleacetic acid (IAA), indole butyric acid (IBA), gibberellin (GA), methyl jasmonate (MeJA), and zeatin nucleoside (ZR) were higher in mycorrhizal plants under DS conditions than the levels in non-mycorrhizal plants (Zhang et al., 2017; Al-Arjani et al., 2020), which promoted both the growth of plants and mycorrhizae. AM fungi dramatically elevated IAA, MeJA, and salicylic acid (SA) content in tomato plants under DS conditions, relative to non-AM plants (Table 1; Sanchez-Romera et al., 2018). Recently, Quiroga et al. (2020b) observed that IAA was involved in radial water transport in AM plants under DS through regulating *AQPs* expression. ABA is a key signal molecule in roots and the production of ABA is essential for the colonization of AM fungi (Herrera-Medina et al., 2007). There is evidence that ABA has an impact on the development and function of AM fungi, so the increase in ABA content in AM plants under DS conditions may stimulate AM development, which would be contribute to enhanced drought tolerance of the plants (Herrera-Medina et al., 2007; Pozo et al., 2015). In addition to regulating ABA levels in host plants, fungi, including AM fungi, also produce ABA (Esch et al., 1994)., AM fungi increase ABA biosynthesis under DS conditions, thereby, increasing the ABA content in host plants, which would further promote stomatal closure and reduce water loss caused by transpiration (Chitarra et al., 2016). ABA content in host plants, however, has also been reported to be reduced by AM fungi under DS conditions (Kandowangko et al., 2009; Sanchez-Romera et al., 2018; Xie et al., 2018), indicating that the regulation of ABA levels in host plants by AM fungi can vary. AM inoculation of tomato induced the expression of *9-cis-epoxycarotenoid dioxygenase* (*NCED*) in roots under DS conditions, thus, promoting the synthesis of ABA in roots, which resulted in enhanced drought tolerance (Aroca et al., 2008). Alternatively, the ABA content in the roots of AM plants can be reduced, and the signaling pathway for ABA can be altered (Xie et al., 2018). The reduction in ABA content in AM plants under DS conditions has been associated with the development of AM fungal mycelia (Kandowangko et al., 2009; Goicoechea et al., 2010).

### Arbuscular Mycorrhizal-Mediated Gene Expression

Drought stress-induced genes and compounds can be divided into two categories: functional genes, which directly play a role in environmental stress, such as aquaporin (AQP), late embryogenesis abundant (LEA) proteins, sugar, proline, etc., and regulatory genes, which are involved in signal transduction and the regulation of gene expression, including stress-related transcription factors and signal molecules, such as calmodulin-binding protein (Shinozaki and Yamaguchi-Shinozaki, 2007). AM fungi can trigger the expression of host stress-related genes under DS conditions (Table 1; Li and Chen, 2012). AM fungi impact the transmembrane transport of water by regulating *AQP* genes that encode aquaporin water-channel proteins located on cell membranes, which may be one of the mechanisms by which mycorrhizal fungi enhance drought tolerance in plants (Rapparini and Peñuelas, 2014; He et al., 2019; Cheng et al., 2020). A total of six fungal AQP proteins including GintAQP1 in *G*. *intraradices*, GintAQPF1 in *G*. *intraradices*, GintAQPF2 in *G*. *intraradices*, RcAQP1 in *Rhizophagus clarus*, RcAQP2 in *R*. *clarus*, and RcAQP3 in *R*. *clarus*, were identified (Aroca et al., 2009; Li et al., 2013; Kikuchi et al., 2016). Drought treatment did not alter *GintAQP1* expression, whereas induced *GintAQPF1* and *GintAQPF2* expression; *RcAQP3* expresses in intraradical hyphae to transport water. Studies have demonstrated that the expression of *plasma membrane intrinsic protein* (*PIP*) genes in *Glycine max* and *Lactuca sativa* plants inoculated with AM fungi was down-regulated under adequate soil moisture conditions (Porcel et al., 2006). In contrast, the inoculation of plants with AM fungi have been shown to increase the expression of *PIPs* in host plants under DS conditions (Zézél et al., 2008). Aroca et al. (2007) found that *PIP* gene family was expressed differently in response to various stresses, depending on the presence or absence of AM fungi. The expression of two functional genes encoding *AQPs* in both drought-exposed maize roots and AM fungi have been shown to be elevated in inoculated plants, relative to non-inoculated plants, indicating that AM fungi simultaneously regulate the expression of *AQPs* in both the host plant and endogenously (Li et al., 2013). In addition, AM fungi down-regulated the expression of seven *PIP* genes, four *nodulin-26 like intrinsic protein* (*NIP*) genes, and six *tonoplast intrinsic protein* (*TIP*) genes under DS conditions while maintaining high-water uptake by mycorrhizal extraradical hyphae (Zou et al., 2019). These results suggest that the regulation of host aquaporins by mycorrhizal fungi is supplemental to hyphal water absorption. A further study revealed that the expression of *AQPs* in host plants was also up-regulated by AM fungi in response to salt stress, while expression levels were diversely affected under well-watered conditions, and marginally or not at all affected under waterlogging conditions (Cheng X. F. et al., 2021). Moreover, the regulation of AQP genes in host plants by AM symbiosis depends mainly on irrigation conditions and the severity of soil drought (Bárzana et al., 2014). Therefore, it appears that AM fungi regulate the expression of host *AQPs* in a manner that is dependent on the type of abiotic stress. Quiroga et al. (2017) observed that AM symbiosis in drought-sensitive varieties regulated *AQPs* more extensively and differentially than in drought-tolerant varieties of maize, suggesting that AM-regulated *AQPs* play an important role in water homeostasis or transport of solutes under DS. Inoculation of corn plants with *R*. *irregularis* increased the phosphorylation status of *PIP2* aquaporins under DS conditions, indicating higher water channel activity in AM plants exposed to DS (Quiroga et al., 2019). Endogenous ABA levels in host plants also affect the impact of AM fungi on *AQP* expression (Ruiz-Lozano and Aroca, 2010b). In addition, SA regulation of plant water conductivity may be related to root *AQP* expression pattern (Quiroga et al., 2018). Two opposing views exist on the mechanism of *AQP* induction in AM plants under DS conditions. The first suggests that an increase in water permeability due to the up-regulation of *AQPs* which would improve the water absorption capacity of plant roots and promote water transport. The second view suggests that the down-regulation of *AQPs* would reduce membrane permeability, thus, preventing cell water loss (Ruiz-Lozano and Aroca, 2010b; Cheng et al., 2020). There is a certain compensation mechanism between the expression of the aquaporin gene *GintAQP1* of AM fungus (e.g., *G*. *intraradices*) and the expression of the host root *AQP*s (Aroca et al., 2009). TcAQP1 in mycorrhizal fungus (*Terfezia claveryi*) is a fungal major intrinsic protein with the function of water and CO_2_ transport, and TcAQP1 has high water conductivity to adapt to DS (Navarro-Ródenas et al., 2012). The increase in the expression of *LbAQP1* of *Laccaria bicolor* requires the contact between mycorrhizal fungi and roots within a short time, and LbAQP1 promotes the transport of NO, H_2_O_2_, and CO_2_ when it is expressed heterologously in yeast (Navarro-Ródenas et al., 2015). It suggests that the AQPs of the host and mycorrhizal fungus may be involved in the transport of water and solutes after drought induction, thus, contributing to the drought tolerance of AM plants.

*LEA* and proline synthesis enzyme Δ^1^-pyrroline-5-carboxylate synthetase (*P5CS*) gene expression is associated with AM-enhanced drought tolerance in plants (Zheng et al., 2020). LEA proteins play a role in reducing water loss and act as molecular chaperones that increase drought tolerance (Ali et al., 2020a). Studies have shown that AM fungi increase the accumulation of dehydrins, a type of LEA protein, in plants, thereby, playing an important role in improving plant drought tolerance (Ruiz-Lozano et al., 2008). AM fungi also induces the expression of *P5CS* in host plants under abiotic stress conditions (Zheng et al., 2020). Cheng H. Q. et al. (2021) identified and cloned an H^+^-ATPase gene, *PtAHA2*, from trifoliate orange and reported that *PtAHA2* expression was up-regulated by both DS and AM fungi inoculation. The up-regulation of *PtAHA2* by mycorrhization further triggered an increase in ammonium nitrogen content in roots and an increase in soil acidification. Huang et al. (2021a) cloned a *MdIAA24* gene from apple and found that overexpression of *MdIAA24* enhanced drought tolerance, as evidenced by a higher level of ROS scavenging, a greater osmotic adjustment ability, improved gas exchange capacity, and increased chlorophyll fluorescence, by regulating AM colonization and arbuscule numbers, as well as an increase in the level of strigolactone. Interestingly, the combination of AM fungi and DS had a synergistic effect on the up-regulation of *malectin-like domain-containing receptor-like kinases* (*MRLKs*) (Luo et al., 2020). A synergistic effect of AM fungi and DS was also observed on the up-regulation of *calcium-dependent protein kinases* (*CDPKs*) in citrus, where 17 *CsCDPK* family members were induced, and *CsCDPK20* and *CsCDPK22* expression was up-regulated in AM plants under DS conditions, relative to well-watered conditions (Shu et al., 2020b). DS and AM fungi colonization collectively induced *calcineurin B-like protein* (*CBL*) *7* and *CBL-interacting protein kinase* (*CIPK*) *4* expression in *Citrus sinensis*. Notably, *CsCBL* and *CsCIPK* exhibited a co-expression pattern in response to DS and AM fungal colonization, as evidenced by the positive correlation of *CsCBL1* expression with *CsCIPK1*, *3*, *6*, and *9* expression (Shu et al., 2020a). Transgenic apple plants overexpressing *MdGH3-2* and *MdGH3-12* and colonized by AM fungi exhibited a greater sensitivity to drought stress than wild type plants, suggesting that *MdGH3-2/12* plays an important role in regulating drought tolerance in apple (Huang et al., 2021b). The regulation of stress-related gene expression and physiology in host plants by AM fungi has been demonstrated to enhance drought tolerance in host plants. The molecular regulatory network associated with mycorrhizal colonization and DS has not been fully elucidated, although the role of several genes has been identified and analyzed.

## Conclusion and Future Prospects

Drought stress causes a significant reduction in plant growth and yield. AM symbiosis with host plants has been shown to play a positive role in mitigating drought damage. AM fungi construct some of their own water uptake mechanisms and also rapidly activate the host’s physiological, molecular and morphological responses to DS that enhances their ability to cope with the adverse effects of drought (Figure 1), increasing the ability of host plants to survive and maintain vigorous growth under drought conditions. Hence, the plant–AM fungi interaction represents a great example of a sustainable agricultural strategy. Notably, the use of biochar amendments further increases AM-mediated drought tolerance in host plants (Hashem et al., 2019). The role of AM fungi in enhancing plant drought tolerance has been extensively demonstrated experimentally, however, the mechanisms involved are very complex and need to be further explored and elucidated. Future work should focus on the following topics:

(1) Studies have demonstrated water absorption by mycorrhizal extraradical hyphae and subsequent transfer to host cells. The mechanism by which extraradical hyphae absorb water and arbuscules unload the water absorbed by the mycorrhizal hyphae, however, is very complex and require further study. Ezawa and Saito (2018) proposed a model on how water and Pi transport may be linked and how fungal aquaporins (AQP3) may participate in the water transport at the intraradical hyphae. How do Pi and water interact in such a complex process? Who is dominant? What specific genes are involved in the offloading of roots from environment to arbuscule-contained root cells?
(2) The benefit of AM fungi is more pronounced under drought conditions than under adequate water conditions. Why does this benefit occur? By what mechanisms does this mycorrhizal benefit occur? Additionally, since AM fungi contribute to enhanced drought tolerance in host plants, they should be utilized in the revegetation of degraded woodlands, farmlands, and pastures in arid and semi-arid areas (Nouri et al., 2020). Further studies in this area are highly warranted.
(3) AM fungi respond to drought by regulating a variety of metabolites, including PAs, FAs, proline, betaine, and osmoregulators in host plants. Studies focusing on a single metabolite cannot fully elucidate its role in drought tolerance. Additional studies are needed to reveal the impact of AM colonization on metabolomic changes in response to DS conditions. Studies of both the non-targeted metabolome and the targeted metabolome are needed.
(4) Studies of the transcriptome in AM versus non-AM plants under DS conditions will provide further identification of AM fungal-specific gene regulation and how these genes are regulated by AM fungi. Overexpression or silencing of specific identified genes from AM fungi and/or the host plant will help to elucidate the mechanisms by which AM fungi enhance drought tolerance.
(5) Whole genome sequences of individual AM fungal strains (e.g., *R*. *irregularis*) have been published (Tisserant et al., 2013), and some stress-related genes encoded by AM fungi have been identified. The mechanisms by which these genes regulate stress response should be further explored.

## Author Contributions

All authors listed have made a substantial, direct, and intellectual contribution to the work, and approved it for publication.

## Conflict of Interest

The authors declare that the research was conducted in the absence of any commercial or financial relationships that could be construed as a potential conflict of interest.

## Publisher’s Note

All claims expressed in this article are solely those of the authors and do not necessarily represent those of their affiliated organizations, or those of the publisher, the editors and the reviewers. Any product that may be evaluated in this article, or claim that may be made by its manufacturer, is not guaranteed or endorsed by the publisher.
